# ^188^Re(V) Nitrido Radiopharmaceuticals for Radionuclide Therapy

**DOI:** 10.3390/ph10010012

**Published:** 2017-01-19

**Authors:** Alessandra Boschi, Petra Martini, Licia Uccelli

**Affiliations:** 1 Department of Morphology, Surgery and Experimental Medicine, University of Ferrara, Ferrara 44121, Italy; petra.martini@unife.it (P.M.); licia.uccelli@unife.it (L.U.); 2 Italy and Legnaro National Laboratories, Italian National Institute for Nuclear Physics (LNL-INFN), Legnaro (PD) 35020, Italy

**Keywords:** rhenium-188, theranostics, radiopharmaceuticals, radionuclide therapy, peptides, bioconjugates

## Abstract

The favorable nuclear properties of rhenium-188 for therapeutic application are described, together with new methods for the preparation of high yield and stable ^188^Re radiopharmaceuticals characterized by the presence of the nitride rhenium core in their final chemical structure. ^188^Re is readily available from an ^188^W/^188^Re generator system and a parallelism between the general synthetic procedures applied for the preparation of nitride technetium-99m and rhenium-188 theranostics radiopharmaceuticals is reported. Although some differences between the chemical characteristics of the two metallic nitrido fragments are highlighted, it is apparent that the same general procedures developed for the labelling of biologically active molecules with technetium-99m can be applied to rhenium-188 with minor modification. The availability of these chemical strategies, that allow the obtainment, in very high yield and in physiological condition, of ^188^Re radiopharmaceuticals, gives a new attractive prospective to employ this radionuclide for therapeutic applications.

## 1. Introduction

The increase of malignant tumors has led to a dramatic surge in the development of new clinical procedures for cancer treatments, including the radionuclide based therapies employed in nuclear medicine. Ionizing radiation is commonly used by external radiotherapy of cancer patients. In this approach, only a limited area around the primary tumor is treated through irradiation with high energy X rays. Targeted radionuclide therapy, instead, is a systemic treatment that involves the injection of radioactive substances (radiopharmaceuticals) into the blood circulation [1]. The radiopharmaceuticals suited for this purpose are vehicle molecule radionuclide constructs with high tumor affinity, which can transport toxic doses of radiation to tumors and metastases. The tumor specificity of the vehicle molecule is determined by its affinity to target structures, whereas the ionizing radiation emitted by radionuclides is used to kill cancer cells. An ideal radiopharmaceutical for therapeutic purposes must have high specificity and act exclusively in the malignant tumors cells; must have accurate targeting capacity to reach all the tumor cells wherever they are localized; bring maximum doses of radiation to the tumor leaving healthy tissues and organs unscathed; eliminate malignant tumor cells with great effectiveness. This last point, essentially, depends on the radio physical properties and the ionizing radiation emitted by the radionuclide [2]. While imaging procedures in nuclear medicine require radionuclides that will emit γ radiation that can penetrate the body, a different class of radionuclides possessing optimal relative biological effectiveness (RBE) is needed for radionuclide therapy. The RBE depends on linear energy transfer (LET), which is defined as the amount of energy transferred to the material penetrated by an ionizing particle per unit distance (measured in keV·µm^−1^). In principle, the most suitable radionuclides for tumor therapy are those emitting radiation with short penetration into the tissue, high LET and RBE, such as α emitters or nuclides producing the Auger effect. These will also cause more intense radiation induced side effects on accumulation outside the tumor. Moreover, β emitting radioisotopes with their directly ionizing electron radiation, still offer a higher LET and RBE than γ radiation and represent an acceptable compromise between therapeutic efficacy and levels of adverse side effects (Table 1).

For several years, the development of therapeutic radiopharmaceuticals has been limited by the availability of suitable β emitter radionuclides, and even now, most therapeutic procedures are based on the use of radiopharmaceuticals containing iodine-131, yttrium-90. Samarium-153, a mixed emitter of β; γ radiation and strontium-89, a pure β emitter, are used for palliative treatment of bone metastases. Another highly promising radionuclide is lutetium-177, which is gradually replacing yttrium-90 for modern peptide based radio receptor therapy [5,6].

In the last years, the increased knowledge of radiochemical procedures has prompted the possibility to develop new radiopharmaceuticals preparation strategies for therapy using radio- metals with peculiar radionuclides properties [4]. The availability of therapeutic radionuclides with high specific activity and high radionuclidic and chemical purity is important for radionuclide therapy applications. In this context, ^188^Re is a radionuclide with excellent properties that can be used to label many therapeutic molecules, including small molecules, peptides and despite its short half-life, monoclonal antibodies [7]. In particular, the short half-life of rhenium-188 would seem an unfavorable characteristic for therapeutic applications in view of the fact that a radionuclide with long physical half-life will deliver more decay energy than one with a short half-life having the same initial activity and biokinetics in the target tissue. Conversely, it was reported that higher dose rates delivered over shorter treatment times in biological tissues are more effective than lower dose rates delivered over longer periods [8]. Thus, a radionuclide with a shorter half-life will tend to be more biologically effective than one with similar emission energy but with a longer half-life; both have the same initial activity and biokinetics in the target tissue. In this context, rhenium-188 has the advantage over lutetium-177 and iodine-131 during the first day of a treatment, and it has been rated that 5 days after, the total dose delivered to the target by these radionuclides is quite similar [8]. Moreover, patients could be undergoing several treatment cycles until the absorbed dose is high enough for a therapeutic effect. Finally, in order to improve the pharmacokinetic and dosimetric properties of ^188^Re, labeled big biological substrates such as antibodies, the use of locoregional applications and the pretargeting approaches can be used [8].

One potential advantage of rhenium-188 over other therapeutic radionuclides, such as yttrium-90, lutetium-177 and copper-67, is its routine availability because of the ^188^W/^188^Re generator. Nevertheless, preclinical and clinical studies have demonstrated favorable pharmacokinetic and dosimetric properties for several ^188^Re-based therapeutics; the simpler and most known chemistry of yttrium-90, lutetium-177 and copper-67 may be the principal reason for which ^188^Re is less used in clinical practice [9].

Some perspectives and achievements on the radiochemical procedure proposed in the last years for the preparation of very stable high yield ^188^Re radiopharmaceuticals are reported in this review. A special gaze is turned to the description of the synthetic procedures available for the preparation of ^188^Re(V) nitrido radiopharmaceuticals, that for their high stability provide a very attractive outlook for clinical applications.

## 2. Rhenium-188

The β-emitting radionuclide, rhenium-188, is attracting interest as a suitable radionuclide for therapeutic applications due to its peculiar nuclear properties and chemical similarities with the most used diagnostic radionuclide technetium-99m. Rhenium-188 decays through the emission of a high-energy β particle (E_βmax_ = 2.1 MeV) with a half-life of 17.0 h. Associated at this decay mode, there is also a γ-emission of 155 keV that can be conveniently utilized to monitor the course of therapy using a conventional γ camera. Another important advantage comes from the easy availability of this radio-metal, which is available through a transportable ^188^W/^188^Re generator system that offers the prospect of cost-effective radiopharmaceuticals preparation for cancer treatment.

### The Parallelism between Technetium-99m and Rhenium-188 Radiopharmaceuticals Preparation

**Figure 1:**
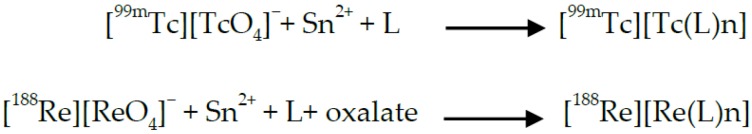
Scheme of the general procedure used for the preparation of high yield ^99m^Tc and ^188^Re radiopharmaceuticals. L represents a particular ligand able to coordinate the metal in a low oxidation state. The addition of oxalate in the ^188^Re procedure allows the preparation of the analogs’ compound in physiological solution.

Technetium and Rhenium belong to the same group 7 of transition metals of the Periodic Table and possess a rich coordination chemistry. The organometallic chemistry of the two elements is well established [10]. In particular, due to the lanthanide contraction, the elements with the atomic numbers 43 (Tc) and 75 (Re), have comparable ionic radii. From the chemical point of view, this means that Tc and Re complexes, prepared by the reaction of the peculiarly coordinating ligands with the transition metal, give rise to analog complexes having exactly the same chemical structure and stability and differ only by the nature of the radio metal center. In these conditions, it is reasonable to expect that these analog complexes of technetium-99m and rhenium-188 have the same biological behavior; a fundamental aspect for theranostic applications in nuclear medicine. The first parallelism between the two radio metals is found in the method of production of the tetraoxo ions [^99m^Tc][TcO_4_]^−^ and [^188^Re][ReO_4_]^−^ based on the use of an alumina column onto which the corresponding parent nuclide, ^99^Mo or ^188^W, is adsorbed as tetraoxo molybdate [^99^Mo][MoO_4_]^2−^ or wolframate anion [^188^W][WO_4_]^2−^ respectively. The radioactive β decay of the ^99^Mo nuclide yields the daughter nuclide ^99m^Tc in the chemical form pertechnetate [^99m^Tc][TcO_4_]^−^ which is successively eluted with a saline solution. This situation parallels completely that of ^188^Re, which is obtained after the radioactive β decay of the ^188^W nuclide and eluted through the alumina column, in the chemical form of perrhenate [^188^Re][ReO_4_]^−^ with a physiological solution. However, due to the low specific activity of ^188^W, the ^188^Re obtained from ^188^W/^188^Re generators is eluted with low radioactivity concentration. For this reason, it often needs an additional concentration step for therapeutic applications [11,12,13,14,15,16]. Alternative pathways such as ^188^W/^188^Re gel generators based on zirconium or titanium wolframate, have also been exploited [17,18], however nowadays most of the commercial ^188^W/^188^Re generators are based on alumina as sorbent on the column. Starting from the above considerations and taking into account the recent progress in the development of the coordination chemistry of technetium, that is directly related with the extended use of ^99m^Tc compounds in diagnostic nuclear medicine [19], it can be expected that the same well established procedure developed for the preparation of ^99m^Tc radiopharmaceuticals (^99m^Tc-RPs) could be applied for the preparation of the corresponding ^188^Re radiopharmaceuticals (^188^Re-RPs). The generator produced [^188^Re][ReO_4_]^−^ represents the starting material for the preparation of rhenium-188 radiopharmaceuticals exactly likewise; [^99m^Tc][TcO_4_]^−^ is the ubiquitous starting material for the preparation of ^99m^Tc-RPs [20,21]. Unfortunately, it was found that the methods utilized for the preparation of high yield ^188^Re-RPs cannot simply follow routes employed for ^99m^Tc-RPs production, and more drastic conditions, such as very acidic pH, high amount of reduction agents and prolonged reaction time, are required [22]. This fact always constitutes a fundamental challenge for the efficient development of new ^188^Re-RPs. The reason for this is the fundamental difference between the values of the standard reduction potentials of the redox reaction involving rhenium and technetium compounds. In particular, E° of a redox process that involves technetium is, on average, 200 mV higher than that of the corresponding rhenium process. This means that, in the same reaction conditions, the perrhenate reduction is more difficult than the pertechnetate one. Recently, a solution to this problem based on the use of an oxalate ion (C_2_O_4_)^2−^ has been proposed [16]. The results demonstrated that the addition of Na_2_C_2_O_4_ to radiopharmaceutical preparations starting from generator-eluted [^188^Re][ReO_4_]^−^ dramatically improved the yield of the final ^188^Re-compound. By the use of the above mentioned strategy, the high-yield production of [^188^Re][ReO(DMSA)_2_]^−^ radiopharmaceutical in physiological conditions was performed [16]. This general procedure allows, theoretically, the application of the standard and well known procedure used for the preparation of ^99m^Tc-RPs to the preparation of analog ^188^Re-RPs with minor modification (Figure 1).

## 3. Design of ^188^Re Radiopharmaceuticals

A fundamental prerequisite for developing a therapeutic agent with some potential clinical utility is to produce a final metallic compound showing high chemical stability and inertness under physiological conditions. The design of a highly stable ^188^Re conjugate can be achieved through a careful selection of the most stable rhenium cores. A rhenium atom tightly bound to some characteristic ligand gains functional groups that usually are strongly resistant to hydrolysis by water molecules. The rhenium(V) nitride, [Re≡N]^2+^ and the rhenium(I) triscarbonyl, [Re(CO)_3_]^+^ cores are among the most stable chemical fragments [23,24,25]. In particular, the [Re≡N]^2+^ constitutes a characteristic functional moiety in which the Re^+5^ ion is multiply bonded to a nitride nitrogen atom (N^3−^). The resulting arrangement of atoms exhibits a very high chemical stability towards both oxidation-reduction reactions involving the rhenium ion and pH variations. This suggests that the synthesis of rhenium(V) nitride radiopharmaceuticals containing the Re≡N multiple bond would allow the facile variation of the other ancillary ligands coordinated to the metal center and hence make possible the fine tuning of the biological properties of the resulting compounds.

### 3.1. The Preparation of ^188^Re(V) Nitrido Radiopharmaceuticals

Starting from the original method developed for the tracer-level preparation of the [^99m^Tc][Tc≡N]^2+^ radiopharmaceuticals [25], the oxalate-based method has been then utilized to develop the first efficient procedure for producing the [^188^Re][Re≡N]^2+^ core from [^188^Re][ReO_4_]^−^, at tracer level and under physiological conditions [26]. The above mentioned approach involves the reaction of the generator eluted [^99m^Tc][TcO_4_]^−^ with a reducing agent and a particular chemical species source of the nitride nitrogen atoms N^3−^, able to produce in solution intermediate nitride ^99m^Tc complexes. This intermediate mixture is converted in a single final product due to the addition of a strong bidentate coordinating ligand. Initially, the method was based on the reaction of [^99m^Tc][TcO_4_]^−^ with [H_2_NN(CH_3_)C(=S)SCH_3_] (DTCZ), that behaves as an efficient donor of nitride nitrogen atoms N^3−^ to yield the [Tc≡N]^2+^ group, in acidic conditions and in the presence of triphenylphosphine. Then, it was observed that the formation of the [Tc≡N]^2+^ group is independent of both the choice of the reducing agent and pH conditions, and that it also takes place at neutral pH using SnCl_2_ as reductant. This method was utilized for the development of bis(dithiocarbamato) nitrido technetium-99m radiopharmaceuticals, a new class of neutral myocardial imaging agents [27,28]. A different donor of nitride nitrogen atoms N^3−^, succinic dihydrazide (SDH), was also used in the lyophilized kit formulation for the preparation of the new cardiac tracer [^99m^Tc]N-DBODC [29].

**Figure 2:**
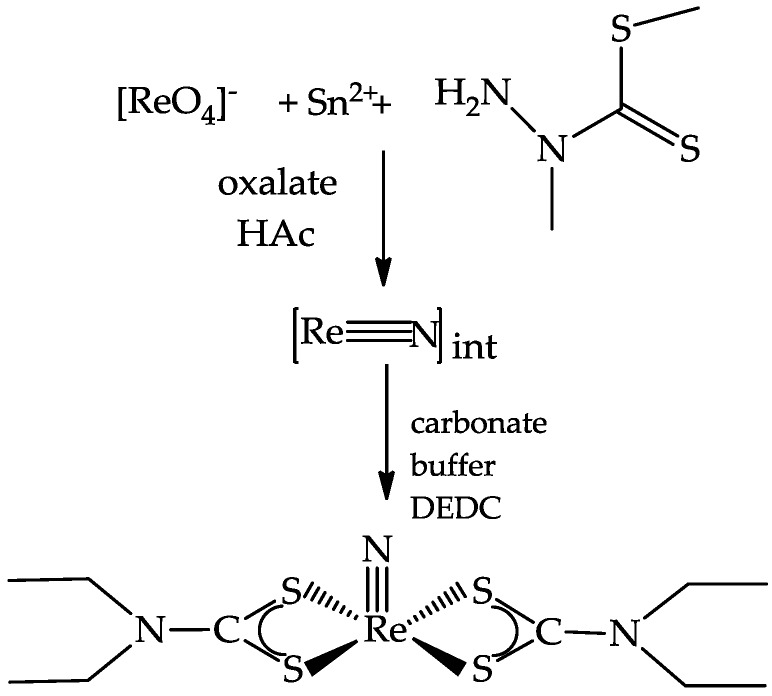
Two step procedure for the preparation of the nitride complex [^188^Re][Re(N)(R_2_NCS_2_)_2_], R = –CH _2_CH_3_.

Starting from these procedures and adding sodium oxalate to the first step of the reaction, ^188^ReN-RPs have been prepared in high yields and in physiological solutions. Bis(dithiocarbamato) nitrido rhenium-188 complexes were carried out using a two-step procedure. In the first step, generator eluted [^188^Re][ReO_4_]^−^ was reacted in the presence of acetic acid with SnCl_2_, oxalate and DTCZ to form nitride ^188^ReN-intermediate complexes with chemical nature not fully determined which, after the addition of carbonate buffer and the corresponding R_2_NCS_2_Na (R= alkyl group) ligand, completely converted in the final nitride complex [^188^Re][Re(N)(R_2_NCS_2_)_2_] (Figure 2).

The highly lipophilic complex bis(diethyldithiocarbamato) nitride rhenium-188, ^188^ReN-DEDC, has been successively utilized for labelling lipiodol an iodinated ethyl ester of the poppy-seed fatty oil for the treatment of the Hepatocellular carcinoma (HCC). HCC is one of the most frequent cancerous diseases in Asiatic and South American areas; it often appears late and successful surgical resection in most cases is difficult or impossible. Carriers of therapeutic agents, such as lipiodol, treat hepatoma without damage to normal tissues. The strategy used for the lipiodol labelling is the dissolution of the strongly lipophilic ^188^ReN-DEDC compound into lipiodol, which constitutes a highly hydrophobic material [30]. Using this strategy, ^188^Re is tightly retained into the fatty oil as a consequence of its strong hydrophobic interaction with the lipophilic metal complex. Similar procedures have been attempted with lipophilic oxo complexes of ^188^Re, but the failure of these methods may be attributed to the difficulty in producing the required lipophilic ^188^Re complex in high yield [31,32,33,34,35]. This result reflects the difficulty in obtaining ^188^Re complexes in satisfactory yield and the intrinsic instability of oxo rhenium compounds in comparison with that of nitride rhenium complexes. Whole-body SPECT images of HCC patients after intrahepatic arterial administration of the nitride ^188^ReN-DEDC labeled lipiodol demonstrated excellent uptake in the lesion without significant activity in the gut and lungs and stable retention of activity in hepatoma was revealed at 20 h after administration with minimal increase in colonic activity and some uptake in the spleen [36].

An automated synthesis module for ^188^ReN-DEDC, labeled lipiodol preparation, has been also reported [37]. The system allows the easy preparation of sterile and pyrogen-free samples of ^188^Re lipiodol ready to be administered to the patient with a dramatic decrease of operator’s radiation exposure. Application of the automated apparatus could be also extended to the preparation of other small therapeutic radiopharmaceuticals molecules labelled with different β-emitting radionuclides.

**Figure 3:**
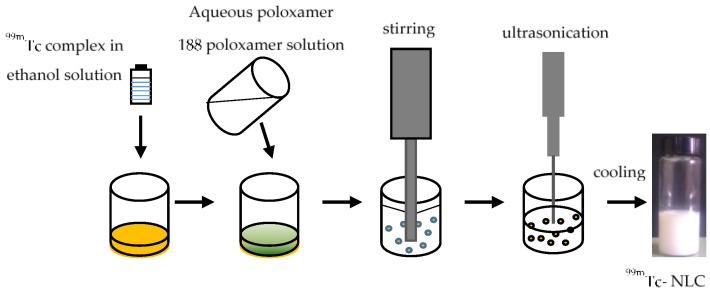
Production of ^99m^Tc-NLC by the 3-step procedure schematized in the figure. The lipid mixture, constituted of the tristearin/miglyol plus ^99m^TcN-DBODC_2_ complex is melted at 80 °C; an aqueous poloxamer 188 solution is added. The two-phase system is high-speed stirred and subsequently ultrasonicated. After cooling at room temperature, the nanoparticle suspension has a homogeneous milky appearance [38].

The strategy used for labelling lipiodol with the ^188^ReN-DEDC compound could also be applied to the labelling of nanostructured lipid carriers (NLC). Recently, a new NLC production protocol has allowed one to firmly encapsulate a lipophilic nitride technetium-99m dithiocarbamate compound, ^99m^TcN-DBODC_2_, within nanoparticles (Figure 3) [38]. In vivo studies have evidenced that NLC remains stable in vivo, suggesting their suitability as a drugs-controlled release system for therapeutic and diagnostic purposes. It is reasonable to assume that similar procedures could be applied to the labelling of NLC with the analog ^188^ReN compound and used in radionuclide-targeted cancer therapy.

### 3.2. The Labelling of Bioactive Molecules with the ^188^ReN Core

In the last years, the labelling of biologically active molecules has become the most efficient tool to generate affinity of ^99m^Tc and ^188^Re complexes for a specific biological target. The most common design of this type of diagnostic and therapeutic agents is based on the so-called “bifunctional approach” [39]. This strategy consists firstly of the selection of an appropriate biologically active molecule having affinity for a specific biological substrate, and the choice of a convenient chelating system that allows one to stably bind the metal. The connection of these two different molecular blocks, which can be realized though a suitable chemical linker, results in a bifunctional ligand. The corresponding conjugate complex, which is formed by the coordination of the bifunctional ligand to the metal, will incorporate the biologically active molecule within its structure as an appended side chain. Various chelate systems of the type “N_2_S_2_” or “N_3_S” have been investigated for the stable labelling of different biomolecules according to the bifunctional approach [40]. Unfortunately, these tetradentate chelating systems rapidly bind the [ReO]^3+^ core to form stable five-coordinate complexes, however the resulting oxo ^188^Re complexes frequently degrade much more rapidly than the technetium analogs, limiting further development of rhenium-based therapeutic agents.

**Figure 4:**
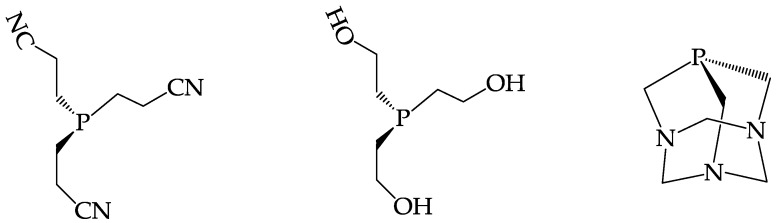
Examples of monophosphine ligands.

In recent years, an alternative approach, called “metal fragment” strategy, to the biologically active labelling, has emerged. The new strategy is based on the preparation of a precursor metal complex, formed by the coordination of specific ligands that stably bind the metal, resulting in a striking inertness towards oxidation-reduction reactions. In addition, this “robust” metal fragment possesses a few substitution-labile coordination positions where transient ligands are easily replaced by a stronger coordination system without reacting with the more stable part of the molecular fragment. If in the chemical structure of the incoming chelating ligand, a bioactive molecule is present, its selective interaction with the metal fragment would result in the formation of a stable final compound incorporating the bioactive substrate. On the basis of this strategy, different technetium and rhenium mixed-ligand compounds have been proposed for the development of target specific radiopharmaceuticals, among which the most representative examples are the [M(CO)_3_]^+^ [41] system, the [M(N)(PNP)]^2+^ ( M = Tc, Re; PNP = phosphinoamine ligand) system [42], and the so-called “3+1” mixed-ligand system [43]. In particular, based on the “3+1” system, a new class of nitride ^99m^Tc and ^188^Re theranostic agents was recently described [43]. These nitrido rhenium radiopharmaceuticals can be prepared following a specific molecular design grounded on the chemical properties of the [M≡N]^2+^ functional group (M = ^188^Re, ^99m^Tc). This group exhibits a predictable chemistry characterized by different structural arrangements whose formation is strongly controlled by the chemical nature of the coordinating ligands. In particular, the chemical characterization of technetium and rhenium nitrido complexes carried out at macroscopic level showed a highly stable arrangement provided by the combination of the π-donor ligand having the set of [X, Y, Z] as coordinating atoms and a monodentate π-acceptor monophosphine ligand (PR_3_) coordinated around the [M≡N]^2+^ (M = ^188^Re, ^99m^Tc) core [44]. This combination, commonly dubbed as “3+1 complexes” has also been observed in mono oxo complexes [45,46,47], however, with the metal nitrido core, the chemical requirements are structurally more restrictive because only a selected set of coordinating atoms can give rise to this molecular arrangement. The essential point here is that the tridentate XYZ ligand should be composed by a combination of three donor atoms that have to be selected from the restricted set of S and N donor atoms. On the contrary, the monodentate ligandhas to be selected from the category of the π -acceptor ligand, e.g., the monophosphines. A list of a monophosphines that are commercially available or can be easily prepared are reported in Figure 4.

**Figure 5:**
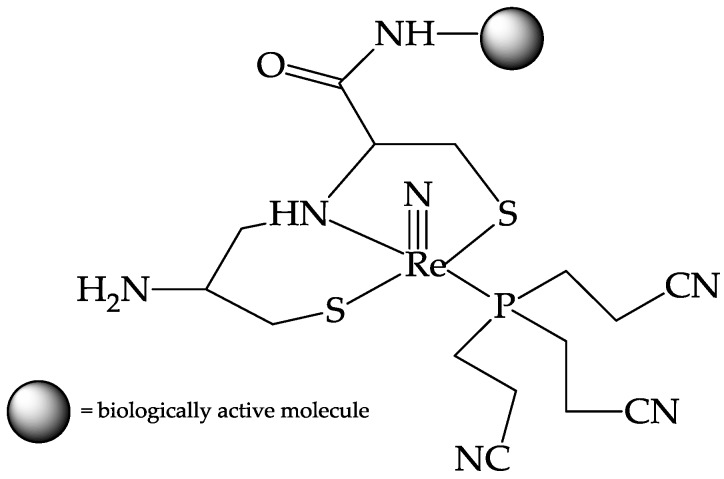
Schematic nitrido ^188^Re “3+1” complex. The circle represents a general bioactive molecule that may be chemically conjugated to the 𝜋-donor SNS-type ligand.

The highest structural stabilization was achieved when these two different types of ligands were simultaneously added to the intermediate [^99m^Tc][TcN]^2+^ fragment where the resulting “3+1” coordination environment was exclusively formed. On the other hand, marked differences were observed with ^188^Re-nitrido complexes for which the same set of coordinating atoms was not as selective as that for analog nitrido ^99m^Tc complexes. In particular, reactions performed with a monothiol ligand, such as 2-mercaptoethanol (HS = HSCH_2_CH_2_OH), revealed once again some differences between the chemical characteristics of the two metallic nitrido fragments, in contradiction to the conventional view that these two elements always exhibit superimposable properties. Indeed, when HS was used as coligand in place of a monophosphine ligand, monoanionic ^188^Re complexes of the type [^188^Re][Re(N)(SNS)(S)]^−^ were isolated in satisfactory yields. On the contrary, no ^99m^Tc complexes of the same type were obtained under similar experimental conditions. The proposed structure for [^188^Re][Re(N)(SNS)(S)]^−^ complexes comprises a SNS-type ligand in the usual tridentate coordination arrangement around the [^188^Re][ReN]^2+^ core, and a monodentate thiol ligand bound to the same group through the deprotonated thiol sulfur atom. Despite these differences, the so-called “3+1” strategy may provide a convenient route to the labelling of bioactive molecules with the [^188^Re][ReN]^2+^ core. In particular, the same SNS-type ligand can be easily prepared by the combinations of two basic amino acids or pseudoamino acids such as cysteine–cysteine, cysteine–isocysteine or cysteine–mercaptoacetic acid to yield potential tridentate chelating systems having [S, N, S] as a set of 𝜋-donor atoms [43]. This tridentate model peptide provides an almost ‘natural’ and straightforward method for labelling peptides with ^188^Re since it only requires lengthening of the original peptidic sequence with a pair of these chelating amino acids and it also provides a convenient substrate for conjugation of biomolecules and pharmacophores to the metallic nitrido fragment (Figure 5).

Smilkov et al. [48] applied the nitride rhenium [^188^Re][ReN]^2+^ “3+1” strategy to label the undecapeptide substance-P (SP), an endogenous ligand for the NK1 receptor type, that plays an important role in modulating pain transmission and may be also involved in the pathogenesis of inflammatory diseases [49]. The presence of functional NK1 receptors has been documented in malignant brain tumors of glial origin, medullary thyroid cancer, non-small cell lung cancer and pancreatic carcinoma and targeting specific SP-NK1 receptors might provide a potential strategy for developing a new radionuclide-based anti-cancer therapy [50]. Based on this consideration, the complex [^188^Re][Re(N)(Cys-Cys-SP)(PCN)], where PCN is (triscyanoethyl)phosphine, has been successfully prepared by applying the “3+1” strategy using the [^188^Re][ReN]^2+^ metal fragment. Incubation of ^188^Re-SP radioconjugate with U-87 MG cells resulted in a pronounced cell surface binding, which reflects the expression level of NK1 receptors on these cells, thus suggesting the affinity of the ^188^Re-compound for the surface membrane receptors.

**Figure 6:**
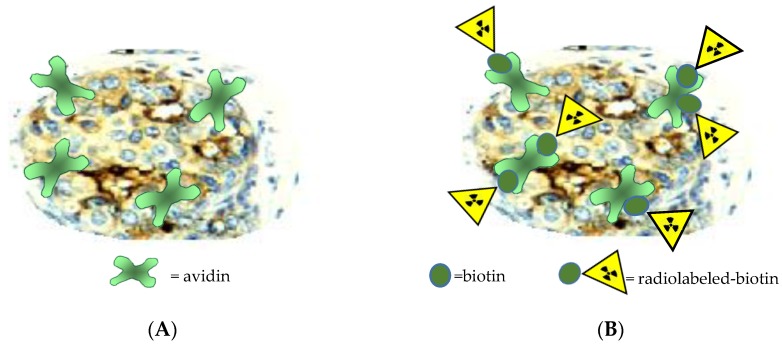

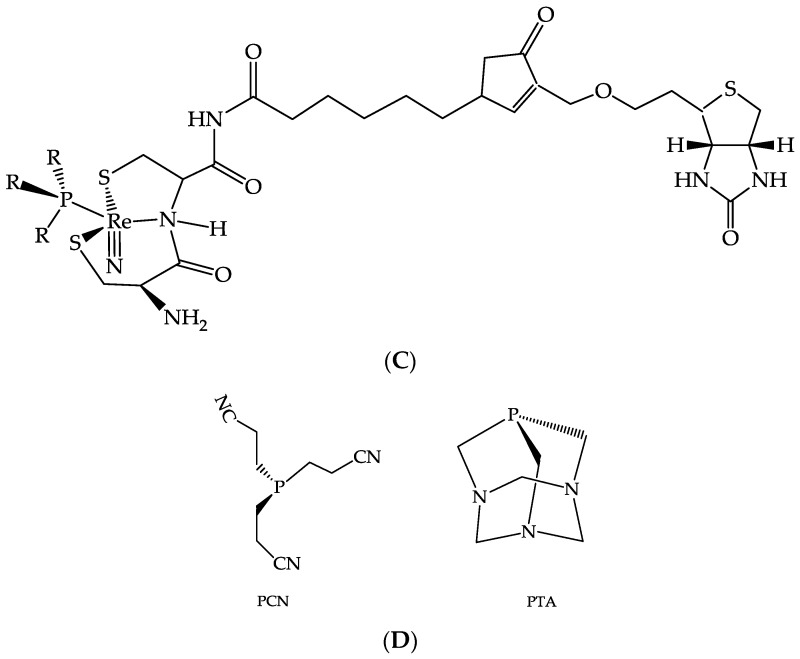
(**A**) Uptake of avidin by tumor cells (schematic representation). Avidin is a charged protein and is avidly taken up by tumor cells because these latter have a strong electrical gradient across their membranes. Avidin is administered in situ by the surgeon after removal of the primary tumor [51]; (**B**) Interaction of radiolabeled biotin with avidin. The subsequent injection of radiolabeled biotin intravenously 16–48 h after surgery led this compound to reach tumor cells and to interact specifically with avidin deposited on the outer membrane; (**C**) ^188^ReN-biotin “3+1” compounds; (**D**) (triscyanoethyl)phosphine (PCN) and 1,3,5-triaza-7 phosphaadamantane (PTA).

The [^188^Re][Re≡N]^2+^ core has been also selected as a basic functional motif for the preparation of biotinylated ^188^Re derivatives for the Intra Avidination for Radionuclide Therapy (IART) approach [51]. IART is a therapeutic approach recently proposed for the treatment of residual malignancies after surgical removal of a primary breast cancer lesion. This therapeutic strategy involves local deposition of avidin in the surgical bed followed by intravenous injection of labeled biotin that is, subsequently, captured by cancerous cells after selective uptake of avidin (Figure 6).

The IART procedure, that consists of a three steps protocol, can be summarized as follows: (1) In the operating theatre, after tumor resection, the surgeon injects directly into the tumor bed 100 mg of avidin diluted in saline; (2) from 16 to 48 h later, 20 mg of biotinylated human serum albumin (HAS) is intravenous (i.v.) injected to mop up any circulating avidin before administration of the radiolabelled biotin; (3) after 5–10 min, radiolabeled biotin is injected i.v. Several studies attempting to prepare ^188^Re biotin derivatives, and various publications on this subject have previously appeared in the scientific literature [52,53,54,55]. However, none of these works reported convincing evidence that the chemical procedures employed were able to afford a stable ^188^Re biotin conjugate in high radiochemical yield (>98%). This result is of extreme importance to avoid unnecessary and dangerous radiation burden to the patient caused by radioactivity not associated with the required chemical form. For this aim, a class of “3 + 1” complexes composed of an apical ^188^Re≡N group surrounded by a tridentate dianionic ligand possessing the 2,2’-dimercapto diethylamine (SNS){[S(CH_2_)_3_NH(CH_2_)_3_S]^−2^} set of coordinating atoms and a monodentate tertiary phosphine ligand (PR_3_) spanning the four coordination positions on the basal plane of a distorted square pyramidal geometry, has been prepared [51,56]. The SNS-type tridentate chelating system utilized was obtained by tethering two cysteine residues (cys–cys), as shown in Figure 6C, whereas the monodentate phosphines were (triscyanoethyl)phosphine (PCN) and 1,3,5-triaza-7 phosphaadamantane (PTA) Figure 6D. The resulting compounds exhibited high in vitro stability and in vivo inertness towards biotin degradation enzymes. Binding affinity of these derivatives towards avidin was determined in vitro. Data indicated that affinity remained almost unchanged after labelling with respect to the free biotin. A model experiment aimed at elucidating the in vivo selective uptake by avidin was carried out in mice. This involved the preliminary intramuscular deposition of colloidal particles embedded with avidin, followed by intravenous injection of ^188^Re labelled biotin. Biodistribution and imaging studies clearly showed that labelled biotin selectively concentrates in the area where avidin colloidal particles were previously distributed. A lyophilized ready-to-use kit formulation was successfully developed to allow easy on-site hospital preparation of this new therapeutic agent.

## 4. Conclusions

It is obvious from the previous sections that the development of new chemical strategies to obtain ^188^ReN-RPs in very high yield and in physiological condition gives a new attractive prospective to the use of ^188^Re for therapy. In particular, simple and efficient procedures are available and could be applied for the labelling of biologically active molecules such as peptides, steroids or other receptor-seeking molecules. Furthermore, the possibility of obtaining ^188^Re in-house and on-demand in high specific activity make this radio metal ideal for nuclear medicine application. Finally, the simplicity of the reported chemical procedures suggests that lyophilized ready-to-use kit formulations could be conveniently developed to allow easy on-site hospital preparations of new therapeutic agents.

## Figures and Tables

**Table 1:** Selected β emitters for radionuclide therapy [1,3,4].

Radionuclides	Half-Life (T_1/2_)	E_βmax_ (keV) *	R_βmax_ (mm) #
^67^Cu	61.9 h	575	2.1
^165^Dy	2.3 h	1285	5.9
^166^Ho	28.8 h	1854	9.0
^131^I	8.0 d	606	2.3
^90^Y	64.1 h	2284	11.3
^177^Lu	6.7 d	497	1.8
^32^P	14.3 d	1710	8.2
^186^Re	3.8 d	1077	4.8
^188^Re	17.0 h	2120	10.4
^89^Sr	50.5 d	1491	7.0

* Maximum energy of β particles emitted; # Maximum range of β particles emitted.
